# Neonatal Nonketotic Hyperglycinemia: A Rare Case from Pakistan

**DOI:** 10.7759/cureus.7235

**Published:** 2020-03-10

**Authors:** Taha Bin Arif, Jawad Ahmed, Farheen Malik, Sharmeen Nasir, Taj M Khan

**Affiliations:** 1 Internal Medicine, Dow University of Health Sciences, Karachi, PAK; 2 Pediatrics, Dow University of Health Sciences, Karachi, PAK; 3 Pediatrics, Baylor College of Medicine/Texas Children's Hospital, Houston, USA

**Keywords:** nonketotic hyperglycinemia, glycine cleavage enzyme, autosomal recessive disorder, glycine cleavage system, glycine encephalopathies, neonates, lethargy, poor feeding

## Abstract

Nonketotic hyperglycinemia (NKH) is an autosomal recessive disorder caused by a defect in glycine cleavage enzyme. It leads to the accumulation of glycine in the body tissues, blood, and cerebrospinal fluid (CSF). Most NKH cases are diagnosed during the natal period of life and are fatal if not promptly diagnosed and managed. Here we present a case of a two-day-old child who presented with reluctant feeding and lethargy. She had reduced tone in all four limbs and a Glasgow Coma Scale score of 9. Keeping an infectious etiology in mind, she was started on cefotaxime and amikacin. The patient was shifted to the neonatal intensive care unit; however, no improvement in the patient's condition was seen and antibiotics were changed to linezolid and meropenem along with initiation of acyclovir. The patient's blood and CSF cultures were negative. Serum amino acid chromatography showed elevated levels of glycine, and a diagnosis of NKH was made. The patient was managed symptomatically but expired on the 22nd day of admission. The case highlights the importance of prompt diagnosis and management of aminoacidopathies. Nearly all metabolic disorders have similar clinical presentations, and an early diagnosis can improve the outcome in patients.

## Introduction

Nonketotic hyperglycinemia (NKH) is an autosomal recessive (AR) disorder, in which glycine metabolism of the body is impaired consequently causing a disproportionate increase and accumulation of glycine in all body tissues, including the central nervous system (CNS) [1]. The primary defect lies in the liver enzyme complex, called the glycine cleavage system. NKH is a rare disease with an estimated incidence of 1 per 250,000 [2]. Glycine encephalopathy has been broadly classified into four major forms; neonatal, infantile, transient, and late. Most glycine encephalopathies occur in the neonates. The disease usually manifests itself within the first few days of life with hypotonia, lethargy, seizures, myoclonic jerks, hiccups, and apnea, which if left untreated can lead to death [3]. In some cases, congenital brain anomalies like hypoplastic corpus callosum and retrocerebellar cyst with hydrocephalus have been reported in association [4,5]. NKH has a very poor prognosis, with a high mortality rate of up to 50% during the first week of life [6]. Therefore, we felt it is imperative to report this case, with the intention to broaden the differential of clinicians when a child presents with hypotonia, encephalopathy, and seizures. Here we present a case of a two-day-old female who was brought to the pediatrics emergency department (PED) with a history of reluctant feeding and lethargy.

## Case presentation

A two-day-old female neonate, unvaccinated, was brought to PED of Civil Hospital Karachi, with complaints of reluctance to feed and lethargy for one day. She was neither taking breastfeed nor formula milk. There was no history of fever, fits, vomiting, or diarrhea. She was the third product of consanguineous marriage and was born via elective cesarean (C-section) at 34 weeks of gestation. The elder two siblings were healthy and alive. No significant family history of miscarriage or stillbirth, chronic disease, or expiry at an early age. Birth history was unremarkable.

On examination, she looked severely lethargic and had a weak cry. She was afebrile with a heart rate of 125 beats/min, respiratory rate of 30 breaths/min, oxygen saturation of 98%, and random blood sugar (RBS) of 80 mg/dL. Anthropometric measurements showed fronto-occipital circumference of 31 cm, length of 42 cm, and weight of 2.1 kg. There were no signs of anemia, jaundice, cyanosis, dehydration, or edema. CNS examination revealed a lethargic child with low Glasgow Coma Scale (GCS) score of 9 with decreased tone in all four limbs, and flat and open anterior fontanelle. Moro, rooting, sucking, and grasping reflexes were poor. The rest of the examinations were unremarkable.

Differential diagnosis of late-preterm with sepsis, meningitis, or encephalitis was established. The patient was initially kept nil per oral and oxygen was provided. She was managed on intravenous (IV) 160 mL 10% dextrose water over 24 hours, IV 160 mg cefotaxime BD, and IV 16 mg amikacin BD. On day 1, the patient developed apnea along with bradycardia and RBS showed a minute increase to 84 mg/dL. She was resuscitated and revived. Aminophylline 12 mg IV stat was given. The patient was shifted to neonatal intensive care unit (NICU), intubated, and put on a ventilator.

The initial investigations (at day of admission) revealed a hemoglobin (Hb) of 17.6 g/dL, mean corpuscular volume of 107 fL, total leukocyte count of 17x10^3^/µL, platelet count (PLT) of 225x10^3^ /µL, and C-reactive protein of 0.5 mg/dL. On the third day, acyclovir was started and antibiotics were changed to meropenem and linezolid as the patient showed no improvement. However, the patient’s GCS remained low (score <8). Ultrasound (US) brain was normal, and CSF detailed report showed protein of 103.3 mg/dL, glucose 95 mg/dL, chloride of 115 mmol/L, and lymphocyte count of 4 with no red blood cells and polymorphs. Urine, CSF, and blood cultures showed no bacterial growth.

On the fourth day, the child’s GCS was still low and pupils became sluggishly reactive with poor reflexes. Non-improvement in the patient’s condition led to the suspicion of a metabolic disorder. Samples were sent to check serum ammonia, arterial blood gases (ABGs), and urinary ketones. On the fifth day, the pupils became mid-dilated and sluggishly reactive to light. Serum ammonia was found to be elevated (443 µg/dL, N<225 µg/dL). ABGs showed mixed severe respiratory and mild metabolic acidosis with an anion gap of 12 and lactate levels of 2.5 mmol/L. Urine was negative for ketones, and RBS was normal throughout the stay. Laboratory investigations throughout the course of stay are summarized in Table 1.

**Table 1:** Laboratory investigations of the patient during the course of hospital stay Hb, hemoglobin; MCV, mean corpuscular volume; TLC, total leukocyte count; PLT, platelets; CRP, C reactive protein; BUN, blood urea nitrogen; Cr, creatinine; Na, sodium; K, potassium, Ca, calcium; T Bil, total bilirubin; PT, prothrombin time; APTT, activated partial thromboplastin time; INR, international normalized ratio

Laboratory Investigation	Normal Value (units)	Day 3	Day 7	Day 13	Day 17	Day 21
Hb	11-15.5 (g/dL)	19.1	14.5	13.5	13.6	12.2
MCV	80-100 (fL)	98	98	92.7	102	106
TLC	4-11 (x10^3^/µL)	13.6	16.3	14.5	17.8	19.4
PLT	140-400 (x10^3^/µL)	187	140	105	14	39
CRP	2-5 (mg/dL)	3.1	41.6	96.6	130	155
BUN	2.5-7.1 (mmol/L)	10	15	11	4	3
Cr	0.3-0.7 (mg/dL)	1.1	1.2	1.0	0.4	0.3
Na	135-145 (mEq/L)	135	142	134	135	140
K	3.0-5.0 (mEq/L)	4.3	3.5	3.1	3.2	3.0
Ca	8.5-10.5 (mg/dL)	7.3	8.3	7.6	8.6	8.1
T Bil	0.3-1.0 (mg/dL)	5.26	9.83	6.19	3.14	3.44
PT	10.5 (seconds)	13.7	14.6	18.2	16.0	15.7
APTT	26-36 (seconds)	30.8	32.2	28.5	29.6	28.1
INR	≤1.10	1.38	1.49	1.93	1.66	1.62

Considering the differential of aminoacidopathies, serum amino acid chromatography and urine organic acid chromatography were ordered. For hyperammonemia, the patient was managed on IV sodium benzoate 500 mg loading dose over two hours with a maintenance dose of 500 mg/day infusion over 24 hours. This regimen was continued for four days, and serum ammonia was repeated which decreased to 226 µg/dL and then 80 µg/dL at which sodium benzoate was stopped.

There was a slight improvement in GCS and respiratory rate during treatment. Attempts of spontaneous breathing trials were failed. PLT and Hb levels dropped and fluctuation was noticed in urea creatinine electrolytes for which the patient was managed accordingly. Attempts to start feeding were done, but the patient could not tolerate more than 5 mL in four hours.

During her stay, the patient developed generalized tonic seizures for which levetiracetam was started. No improvement in GCS was observed even after anti-epileptic therapy (AET). Urine organic acid chromatography was unremarkable. However, serum amino acid chromatography showed severely increased levels of glycine documented about >2,500 µmol/L [N=101-317]. The detailed report of serum amino acid chromatography is shown in Table 2.

**Table 2:** Detailed report of serum amino acid chromatography

Amino Acid	Normal Levels (Units)	Results
Taurine	14-238 (µmol/L)	242
Aspartate	1-21 (µmol/L)	16
Threonine	60-141 (µmol/L)	129
Serine	62-206 (µmol/L)	202
Asparagine	38-114 (µmol/L)	85
Glutamate	32-104 (µmol/L)	133
Glutamine	198-886 (µmol/L)	379
Glycine	101-317 (µmol/L)	>2500
Alanine	108-448 (µmol/L)	452
Citrulline	5-33 (µmol/L)	42
Valine	65-201 (µmol/L)	143
Cystine	20-60 (µmol/L)	38
Methionine	6-50 (µmol/L)	27
Isoleucine	22-82 (µmol/L)	31
Leucine	47-175 (µmol/L)	86
Tyrosine	38-178 (µmol/L)	45
Phenylalanine	21-85 (µmol/L)	57
Ornithine	31-207 (µmol/L)	103
Lysine	67-291 (µmol/L)	337
Histidine	25-113 (µmol/L)	66
Arginine	12-116 (µmol/L)	46
Proline	120-344 (µmol/L)	187

Sodium benzoate was started to reduce serum glycine levels. The patient was diagnosed as a classic neonatal type of NKH which has a very poor prognosis. For further confirmation of diagnosis, CSF glycine levels could not be done due to financial constraints. Another therapeutic option, dextromethorphan (N-methyl-D-aspartate [NMDA] receptor antagonist), was started at 4 mg eight hourly. Unfortunately, the child did not respond to this treatment.

Around the 18th day of admission, the pupils became fixed, dilated, and non-reactive to light. She could not be weaned off from the ventilator. The disease and prognosis of the patient was explained to parents. After a discussion with parents, a do-not-resuscitate code was decided and supportive management was continued. Unfortunately, the patient expired on the 22nd day of admission.

## Discussion

NKH, also known as glycine encephalopathy, is a rare lethal AR metabolic disorder caused by the defect in glycine cleavage enzyme (GCE). Although NKH is uncommon worldwide, it is relatively more common in Finland where it occurs in one in 55,000 newborns [7]. The neonatal type of NKH has a more severe prognosis than late-onset type, and it is characterized by rapid development of neurological symptoms like lethargy, hypotonia, apnea, and seizures. These effects occur due to the accumulation of glycine in different parts of the brain that have glycine receptors. Glycine receptors in the cerebrum and cerebellum have stimulating effects, and accumulation of glycine in these areas leads to the development of seizures, myoclonus, and encephalopathy [8]. Our patient presented with similar complaints of lethargy and reluctance to feed. Clinical examination revealed muscular hypotonia with hyporeflexia and poorly reactive pupils. Later she developed apnea, bradycardia, and generalized tonic seizures not responding to AET.

NKH is primarily diagnosed by determining the glycine levels in plasma and urine. In case of high glycine levels and the absence of ketonemia, the CSF glycine and ratio of CSF glycine to plasma are measured [9]. The definitive diagnosis of NKH is made by measuring the level of GCE on liver biopsy. GCE is a mitochondrial enzyme comprising four different proteins (glycine decarboxylase; P-protein, hydrogen carrier protein; H-protein, aminomethyltransferase; T-protein, and dihydrolipoamide dehydrogenase; L-protein). Any kind of mutation in genes encoding these proteins can lead to NKH [10]. Serum amino acid chromatography revealed an approximately 10-fold increase in glycine levels in our patient. However, CSF glycine and GCE levels were not determined due to a lack of financial support. Similarly, no significant findings were observed in the US brain of our patient which coincides with the fact that imaging studies are not so effective in diagnosing this condition. Considering that the signs and symptoms of this disorder mimic sepsis, encephalitis, or meningitis, the patient was advised for CSF detailed report, blood, urine, and CSF culture. However, no significant bacterial growth was observed in these reports.

Being a fatal disease, NKH has devastating consequences. The majority of patients die within the first week of life and those who survive suffer from severe mental retardation [11]. Seizures in this disorder do not respond to conventional AET, and the patient ultimately dies. It has also been observed that the brain is less affected by the toxic concentration of glycine and develop lesser complications if the condition is diagnosed and managed earlier [12]. In a resource-limited setup, such types of rare conditions are difficult to diagnose and treat which consequently leads to the death of affected patients and a decline in overall prognosis.

The treatment of NKH consists of three drug groups. (1) Plasma glycine-lowering agents like benzoate that reduce the level of plasma and CSF glycine leading to a decrease in seizure frequency. These drugs improve arousal but have no impact on the neurological status of the individual [12-14]. (2) NMDA inhibitors are another group of drugs that control seizures and improve neurological functions by antagonizing the effects of NMDA receptors in the brain. Two important drugs of this group are dextromethorphan, a common antitussive, and ketamine. These drugs particularly improve neonatal swallowing and sucking reflexes and modify abnormal electroencephalographic discharges from the brain [13,15,16]. (3) Competitive glycine inhibitors which include strychnine. These drugs are used to treat mild cases of NKH [16].

Due to the lack of definitive diagnosis at admission, our patient was initially given symptomatic treatment but no improvement by antibiotics and antiviral agents was observed. IV sodium benzoate was administered considering increased levels of serum ammonia and an improvement in GCS was observed for a short period of time. Later the deterioration of condition due to the development of seizures created a diagnostic dilemma. Medication (levetiracetam) was not able to control seizures in this patient. After confirmation of the diagnosis and thorough literature search, the patient was started on sodium benzoate and dextromethorphan. However, a transient neurological improvement was observed after the initiation of treatment with glycine-lowering drugs. Parents were counseled about the poor prognosis of this condition. 

Due to delay in diagnosis and appropriate treatment, there was a high possibility that the CNS of this patient probably had been irreversibly damaged by the toxic accumulation of glycine. Therefore, it is imperative to make a correct diagnosis as early as possible to improve mortality in these patients. This can further aid in genetic counseling and appropriate prenatal diagnosis in subsequent pregnancies. Unfortunately, the patient did not survive due to a lack of prompt management in a resource-limited setting.

## Conclusions

Since most of the clinical features of inborn errors of metabolism coincide with sepsis or other common diseases of the neonatal period, a delay is observed in diagnosis and adequate management of rare cases like NKH. This leads to devastating outcomes as highlighted in our study. It cannot be emphasized enough that early diagnosis and care of neonatal NKH is crucial. In our case, due to lack of prompt investigations and diagnosis, the delay in initiation of treatment led to a steep decline in patient's condition. Despite the putative treatment with sodium benzoate and dextromethorphan, the prognosis of this condition is still grave. Hence, it is important to consider NKH among differential diagnoses of a neonate coming with seizures and altered level of consciousness without distinct acid-base disturbances.
